# Bioeconomy and Climate Change: The Scenarios of Food Insecurity in Brazil’s Northern Region (Amazon) Due to the Shift from Traditional Table Crops to Globally Valued Commodities

**DOI:** 10.3390/foods14234146

**Published:** 2025-12-03

**Authors:** Waldeir Pereira, Tulio Lara, Antônio Andrade, Marcos Seruffo, Aurilene Andrade, Cláudio Silva, Bergson Bezerra, Keila Mendes, Iolanda Reis, Iracenir Santos, Larice Marinho, Hildo Nunes, Juliane Barros, Matheus Lima, Lucas Silva, Roberto Monteiro, José Santos, Theomar Neves, Raoni Santana, Lucas Vaz Peres, Alex Silva, Petia Oliveira, Aldeize Tribuzy, Eliandra Sia, Daniela Pauletto, Celeste Rossi, André Silva, Francisco Silva, Letícia Moreira, Pio Lima-Netto, Celson Lima, Gabriel Brito-Costa

**Affiliations:** 1 Research Group Interaction Biosphere-Atmosphere and Micrometeorology on Amazonia (IBAMA), Federal University of Western Pará (UFOPA), Santarém 68040-255, Brazil; waldeir23santos@gmail.com (W.P.);; 2 Biosciences Post-Graduate Program (PPGBIO), Federal University of Western Pará (UFOPA), Santarém 68035-110, Brazil; 3 Institute of Engineering and Geosciences, Federal University of West Pará, Rua Vera Paz s/n, Santarém 68040-255, Brazil; 4 Anthropic Studies in the Amazon Post-Graduate Program (PPGEAA), Federal University of Pará, Castanhal 68740-222, Brazil; 5 Climate Sciences Post-Graduate Program (PPGCC), Federal University of Rio Grande do Norte, Av. Senador Salgado Filho, 3000, Lagoa Nova, Natal 59078-970, Brazil; 6 Institute of Biodiversity and Forests, Federal University of West Pará, Rua Vera Paz s/n, Santarém 68040-255, Brazil; 7 Postgraduate Program in Natural Resources of the Amazon (PPGRNA), Federal University of Western Pará (UFOPA), Santarém 68035-110, Brazil; 8 PhD Program Society, Nature and Development (PPGSND), Federal University of Western Pará (UFOPA), Santarém 68040-255, Brazil; 9 Postgraduate Program in Forest Science, Technology and Innovation (PPGCTIF), Federal University of Western Pará (UFOPA), Santarém 68035-110, Brazil; 10 Biotechnology and Biodiversity—Bionorte Network (REDE BIONORTE), Federal University of Western Pará (UFOPA), Santarém 68040-255, Brazil; 11 Agrometeorology Laboratory with Bioeconomy Modeling and Environmental Diagnosis (LAMBDA), Federal University of Western Pará (UFOPA), Santarém 68040-255, Brazil

**Keywords:** food security, amazon bioeconomy, climate change, agricultural commoditization

## Abstract

Climate variability directly influences agriculture, especially in a scenario of global change and transition to a sustainable bioeconomy. This study analyzed historical series (1994–2023) of productivity and harvested area of annual crops (corn, cassava, and beans) and perennial crops (pineapple, cocoa, annatto, avocado, and guava), in order to understand the relationship between rainfall, maximum temperature, and agricultural production in northern Brazil. To achieve this, the Augmented Dickey–Fuller (ADF) test was applied to verify the stationarity of the series, and principal component analysis (PCA) was used to identify correlation patterns between climate and production variables. The ADF test showed that annual precipitation is stationary, while maximum temperature is non-stationary, confirming a warming trend. Among the crops, only bean productivity was stationary, albeit at low levels, while corn, cassava, and cocoa showed non-stationary behavior, reflecting technological advances combined with climatic pressures. PCA indicated different responses: corn showed a positive association with temperature, but signs of recent stagnation, whereas cassava and beans depended more on precipitation, demonstrating vulnerability to drought. Among perennials, avocado and guava responded positively to increased temperature, while annatto and pineapple were more dependent on rainfall. Cocoa showed a balanced correlation with both variables. It can be concluded that climate impacts on agriculture are heterogeneous and require specific adaptive strategies. From a bioeconomy perspective, the importance of productive diversification, technological innovation, and public policies aimed at climate resilience and the sustainability of low-carbon value chains is highlighted.

## 1. Introduction

The increase in the world’s population, projected to exceed 9.5 billion by 2050, presents urgent challenges for global food security [1]. A considerable increase in food production is clearly demanded, especially in a context in which growing urbanization, changing consumption patterns, and limited natural resources are increasing the complexity of the challenge [2].

According to the FAO, 70% more food will be required by 2050 to feed the growing global population [1]. However, this increase needs to happen without significantly expanding the cultivated area, given that most of the arable land is already in use or facing environmental degradation [3]. This reality demands an agricultural revolution that incorporates advanced technologies and sustainable practices to maximize productivity without depleting available resources [4].

In this scenario, the Brazilian Amazon is one of the world’s last great agricultural frontiers. With vast areas of arable land, the region plays a strategic role in meeting the growing global demand for food and agricultural commodities [5]. Agricultural expansion in the Amazon, however, often comes at the cost of deforestation, loss of biodiversity, and the displacement of local communities [6].

This reflects a dichotomy between the region’s agricultural potential and the challenges associated with its sustainable development. In addition, the Amazon is full of soils that, although rich in biodiversity, often need to be properly managed to sustain large-scale agriculture, which requires significant investment in technology and research to avoid negative environmental impacts [7].

Climate change is also adding a new level of complexity to agricultural production in the Amazon. Climate models project increases in average temperature, prolonged periods of drought, and more irregular rainfall patterns by the end of the century. These environmental changes could radically disrupt the forest’s CO_2_ sink pattern [8,9,10,11,12]; affect the efficiency of other forested environments that work as a CO_2_ sink [13,14,15,16], as well as the quality of life of its population [17]; and change atmospheric circulation patterns [18].

Projections indicate that the Amazon region will be particularly vulnerable to the impacts of climate change, including rising average temperatures, longer periods of drought, and irregular rainfall [2]. These factors have direct implications for agricultural productivity, especially for crops such as cocoa, corn, beans, and cassava, which play a crucial role in the regional economy and food security [1].

For example, cocoa, a commodity with high economic value, could face serious challenges due to rising temperatures, which could alter flowering and fruiting patterns [19]. Similarly, crops such as pineapple and guava can be affected by longer periods of drought, while corn and beans, staple foods, face risks of reduced productivity due to water variation [20]. These periods of drought tend to be more prolonged and frequent, mainly due to the intensification of the ENOS phenomenon (El Niño Southern Oscillation).

The ENOS phenomenon has a direct influence on atmospheric circulation patterns, altering rainfall patterns in different parts of the world, including the tropics and the extratropics of South America [21,22]. According to the National Oceanic and Atmospheric Administration (NOAA) [23], to be classified as El Niño or La Niña, based on historical climate patterns, the phenomenon must be sequenced for 5 months each quarter. The US National Oceanic and Atmospheric Administration also explains that another indicator for classifying the respective events is the monthly anomalies of the Ñino 3. 4 OISST (OCEANIC Niño Index), when the temperature reaches or exceeds +/−0.5 °C, together with other consistent atmospheric parameters, persisting for up to 3 months in a sequence.

Research [24] has estimated that the Super El Niño that occurred in 2023/2024 would have the potential to have a major impact on global crop yields and international agricultural markets during this period, with the extreme event causing high temperatures, high radiation levels, abnormal rainfall and extreme weather, resulting in low yields for corn, rice and wheat crops worldwide (corn: −2.3%; rice: −0.4%; wheat: −1.4%: −2.3%; rice: −0.4%; wheat: −1.4%), while after the three years of drought, precipitation would potentially increase in North and South America, the world’s largest soybean producing region, and could benefit from an increase in global soybean yields (+3.5%) due to the influence of La Niña.

Among the climatic elements that events such as droughts influence, air temperature is one of the main ones, as far as plant growth is concerned, which ranges from flowering to fruit ripening [25]. This could compromise the food security of several vulnerable regions, such as the Amazon. In addition, family farming, which traditionally supports the production of basic foodstuffs in the region, is losing ground to the production of agricultural commodities aimed at the international market, such as corn and cocoa [26]. This phenomenon reflects a global tendency where the demand for products of high economic value outstrips the production of essential food products [1].

In the Amazon, this translates into the replacement of diversified crops with commodity monocultures, which offer greater financial returns but increase environmental and social vulnerability. Consequently, foods such as beans, manioc, pineapple, and guava face a decline in production, directly affecting the food security of local populations. To mitigate these challenges, technological innovation has played a central role. Technologies such as precision agriculture, the use of remote sensors, and artificial intelligence are enabling more efficient use of land and natural resources [27]. Vertical farming, for example, is emerging as a promising solution as it allows food to be produced in controlled environments with less water and space consumption [28].

Moreover, the development of genetically modified seeds and integrated pest management practices is helping to increase agricultural productivity while reducing environmental impact. Despite this, the adoption of these technologies still has barriers, especially for small farmers, who lack access to finance and adequate infrastructure. Another promising approach is the bioeconomy, which seeks to integrate the sustainable use of biological resources with technological advances to promote economic and environmental development [29].

The Amazon, with its abundant biodiversity, has a unique potential to lead bioeconomy initiatives that value the region’s natural resources while promoting sustainable agricultural practices [30]. As an example, the integration of agro-forest systems and the use of bio-inputs have proven to be effective in recovering degraded soils and increasing agricultural productivity. The bioeconomy can also create new value chains based on products native to the Amazon, such as açaí, annatto, and cocoa, which are in great demand in international markets.

Strategies such as tax incentives for sustainable agricultural practices, investments in research and development, and training programs for small farmers are essential to ensure that the Amazon has a leading role in global agricultural production without compromising its biodiversity and natural resources [31]. Based on this context, this study aims to analyze the historical dynamics of production levels of key Amazonian bioeconomy crops (pineapple, avocado, cocoa, beans, corn, guava, cassava, and annatto) between 1994 and 2023, and to evaluate how climate variability and market forces are reshaping agricultural production in the region.

Specifically, we address the following research questions: (a) how has the production of food crops changed in Northern Brazil in the last 30 years; (b) what role has the climate played in influencing those changes; (c) what are the consequences of the changes for local economies?

This research provides a novel perspective by contrasting climate and economic pressures on Amazonian agriculture, contributing to the debate on food security and sustainable bioeconomy pathways in the region.

## 2. Materials and Methods

### 2.1. Characterization of Study Area

**Figure 1:**
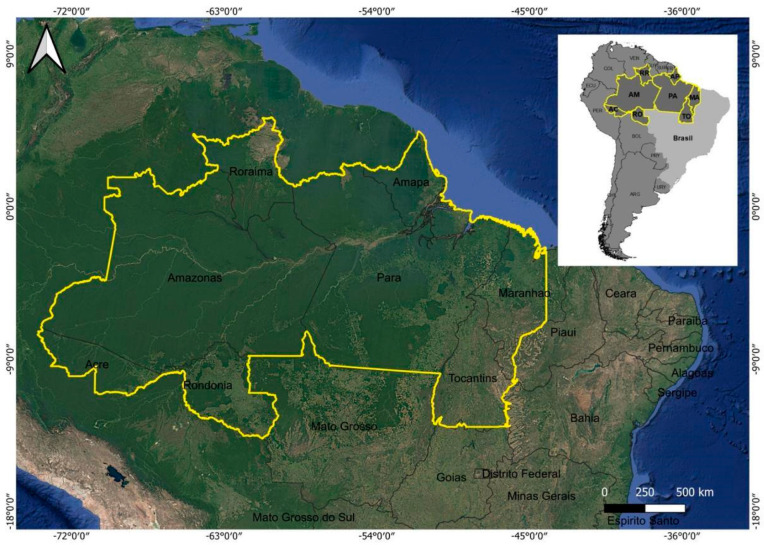
Study area: Northern region of Brazil (Amazon). The yellow polygon indicates the states of Amapá, Amazonas, Acre, Pará, Tocantins, Rondônia, and Roraima.

The study area comprises the northern region of Brazil (the states of Amapá, Amazonas, Acre, Pará, Tocantins, Rondônia, and Roraima), which is part of the Legal Amazon region, and also includes part of the state of Maranhão and the state of Mato Grosso (Figure 1).

The Amazon Basin is often featured in studies on land use change and global climate change, such as studies on carbon (C) cycling and storage and its implications for the global climate [32], besides factors that affect the quality of life of its population [17]. Around 10 to 15% of the earth’s biodiversity is estimated to be found here, which also records abundant rainfall of around 2200 mm/year, making the region an important source of heat and humidity for the atmosphere, generating an estimated 210,000 m^3^·s^−1^ to 220,000 m^3^·s^−1^ of river flow, which is ∼15% of the freshwater input into the oceans, while storing around 150 to 200 billion tons of carbon and also presenting a mosaic of ethnological and linguistic diversity [33].

The northern region of Brazil is one of the largest repositories of biodiversity on the planet, with the Amazon as its main biome. However, it also faces major challenges related to climate change, such as increased deforestation, soil degradation, and biodiversity loss, which have a direct impact on food production, the local economy, and the lives of millions of people who depend on natural resources for sustainable livelihoods. These issues, in a year when the Conference of the Parties (COP)30 was held in the region, bring the spotlight to various aspects of local socio-biodiversity and socio-bioeconomy.

### 2.2. Meteorological Data

Climate Engine is a digital platform that provides data from reanalysis, remote sensing, and meteorological models available on the website (https://app.climateengine.org/), created by a multidisciplinary team of scientists whose main partners are the National Aeronautics and Space Administration—NASA, Google Earth Engine, and the United States Geological Survey—USGS. The service provides a series of products from digital processing, such as maximum air temperature (°C) and precipitation (mm) data from the European Center for Medium-Range Weather Forecasts (ECMWF) ERA5.

Climate Engine is a platform for climate and environmental analysis, using remote sensing and climate data. It enables users to access, visualize, and process satellite and climate information, integrating historical and real-time data for sectors like agriculture, hydrology, and energy. Powered by Google Earth Engine, it supports fast, scalable analysis, generating maps, graphs, and custom reports. The platform offers environmental indices like NDVI (vegetation density) and SPI (drought monitoring), and provides modeled data such as temperature, precipitation, and evapotranspiration from sources like ERA5 and MODIS. By combining spatial and time series analysis, it helps identify trends, assess extreme events, and inform decision-making [34].

In order to have a climatological series, a minimum of 30 years of data was considered, covering the years between 1994 and 2023. The graphs of annual rainfall accumulation and annual maximum air temperature averages (annual averages of maximum daily temperature data) were built in Python 3.0.

### 2.3. Agricultural Production Data

Data were collected from the Brazilian Institute of Geography and Statistics (IBGE) System for the Automatic Retrieval of Aggregated Data (SIDRA). This system provides detailed statistical information on various socio-economic, demographic, and geographical aspects of Brazil. The system allows free access to a large database organized in tables that can be customized according to users’ requirements.

SIDRA provides information from different IBGE surveys, such as the Demographic Census, the National Household Sample Survey (PNAD), the National Broad Consumer Price Index (IPCA), and agricultural production. With interactive tools, users can select specific variables, periods, and regions, generating tables, graphs, and maps adapted to their requests.

The variables related to the annual and perennial crops grown in Brazil each year are identified in this context. Additionally, for this study, data from 1994 to 2023 were considered, taking into account the quantity produced, the area planted, and the area harvested for pineapple, cocoa, beans, guava, manioc, corn, and annatto. These crops were chosen for their importance and prominence in the regional economy, according to the Bioeconomy Plan for the State of Pará (2022) [35].

### 2.4. Statistical Analyses

The analysis of agricultural and climatic time series requires verification of stationarity, since many statistical and econometric methods assume that the data have constant average, variance, and covariance over time. Non-stationary series can lead to spurious inferences, making it difficult to identify causal relationships between variables such as productivity, harvested area, precipitation, and temperature. To assess the stationarity of the series presented in this study (1994–2023), the Augmented Dickey–Fuller (ADF) test, widely used in agricultural and environmental econometrics, was applied. The ADF test is based on the hypothesis that a time series yty_tyt can be represented by an autoregressive process of order [36]. Principal component analysis (PCA) was also conducted, which is a widely used multivariate technique for reducing the dimensionality of data sets and identifying underlying patterns that explain the total observed variability. This study evaluates climate variables (rainfall, maximum temperature) and agricultural variables (productivity) over three decades. Applying PCA allows us to identify which factors have the greatest influence on the joint variability of the agroclimatic system. The relevance of PCA in agroclimatic studies has already been widely highlighted in the literature, being applied both to assess the impacts of climate change on agriculture and to guide public policies and adaptation strategies [37].

## 3. Results

**Figure 2:**
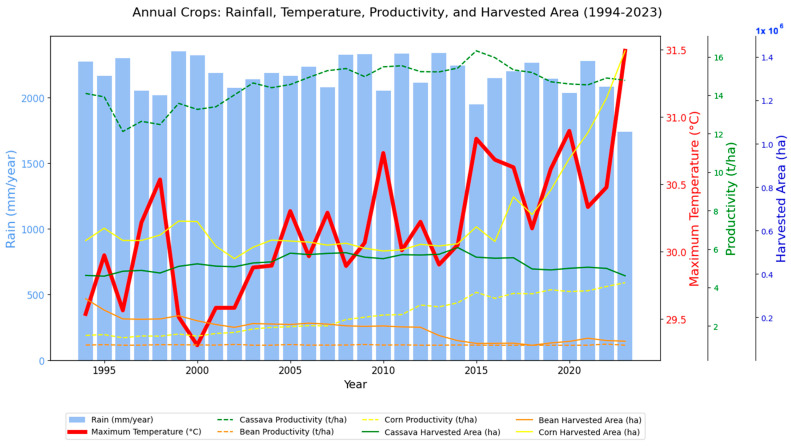
Annual precipitation totals and annual average maximum air temperatures for northern Brazil based on ERA5 data from 1994 to 2023, associated with the productivity of annual crops (corn, cassava, and bean) and the annual harvested area.

Figure 2 summarizes the interaction between climate variables (rainfall and maximum air temperature), agricultural productivity (in tons per hectare), and harvested area (in hectares) for three specific crops—corn, cassava, and pineapple—from 1994 to 2023, characterized as annual crops. The integration of multiple pieces of information allows us to visualize correlations, long-term trends, and possible effects of environmental conditions on agricultural performance.

The left axis, represented in light blue with bars, shows annual precipitation (mm/year). The influence of low-frequency meteorological phenomena, such as ENSO (El Niño Southern Oscillation), is noticeable, causing increased rainfall in the northern region during its cold phase (La Niña) and intense droughts during its warm phase (El Niño). Annual precipitation (total rainfall in mm) varies over the period, with no clear trend of significant increase or decrease, although there are occasional fluctuations, such as reductions in certain years and peaks in others. This stability in precipitation suggests that, at least in the period analyzed, interannual rainfall variability did not significantly compromise total water availability, although minor variations may have a local impact on productivity.

The thick red line shows the average annual maximum temperature (°C), with the axis on the right. Unlike rainfall, temperature shows an upward trend throughout the series. In the early 1990s, values were close to 29.5 °C, with fluctuations, but after 2010, the curve shows a clearer upward trend, exceeding 31 °C in recent years. This increase is consistent with the global trend of climate warming and may have direct implications for plant metabolism, evapotranspiration, and irrigation needs. The maximum air temperature has been increasing over the years, especially since 2005 (one of the years marked by severe droughts in the Amazon region), with an average ranging between 29.5 °C and 31.5 °C.

Corn cultivation shows a clear trend toward increased productivity, reflecting technological investment. In recent years, there has been stagnation, possibly due to limitations imposed by heat. Cassava, on the other hand, maintains stable productivity but is losing harvested area, indicating less economic interest, while bean cultivation maintains low and virtually constant productivity, reflecting climate vulnerability and low technological investment. Cassava and beans have reduced their harvested area over the years, signaling a loss of relative importance in the production system, a factor that raises a warning about a possible increase in food insecurity among local populations.

**Figure 3:**
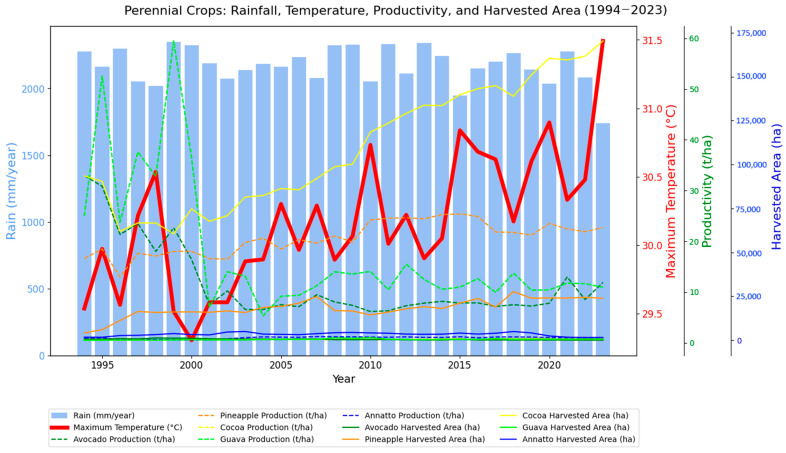
Annual precipitation totals and annual average maximum air temperatures for northern Brazil based on ERA5 data from 1994 to 2023, associated with the productivity of perennial crops (pineapple, avocado, cocoa, guava, and annatto) and the annual harvested area.

Figure 3 shows the data on total rainfall, maximum annual temperature, productivity (t/ha), and harvested area (ha) for four perennial crops: avocado, cocoa, annatto, and guava for the period 1994–2023. As in the previous graph, it is possible to identify relationships between climate and agricultural performance, in addition to the structural trends of each crop over the last three decades. In terms of productivity, there are distinct behaviors among crops. The agricultural productivity (in t/ha) of the crops analyzed shows great interannual variability.

Avocado and guava crops show more marked fluctuations, with no clear trend of growth or decline. In some years, there are significant peaks followed by sharp declines, suggesting strong sensitivity to variable environmental conditions. Avocado production peaked in 1994, when 21,316 tons were produced on 647 hectares harvested (yield of 32.9 t/ha), falling to 767 tons produced on only 78 hectares (yield of 8.5 t/ha) in 2022. while guava did not reach its peak production in 2010 (20,692 tons produced on 1470 hectares, resulting in 14 t/ha), coinciding with peak productivity in 1999 (59.4 t/ha), as this was achieved with a production of 12,137 t on 204 ha, which reinforces the indication of climatic influence on productivity, due to the severe drought of 2010.

Cocoa shows a more stable curve, but also with fluctuations. As a crop typically adapted to shade and humid climates, its productivity may be affected by temperature increases, although the harvested area (yellow line) has grown significantly, which may compensate for losses per hectare.

**Figure 4:**
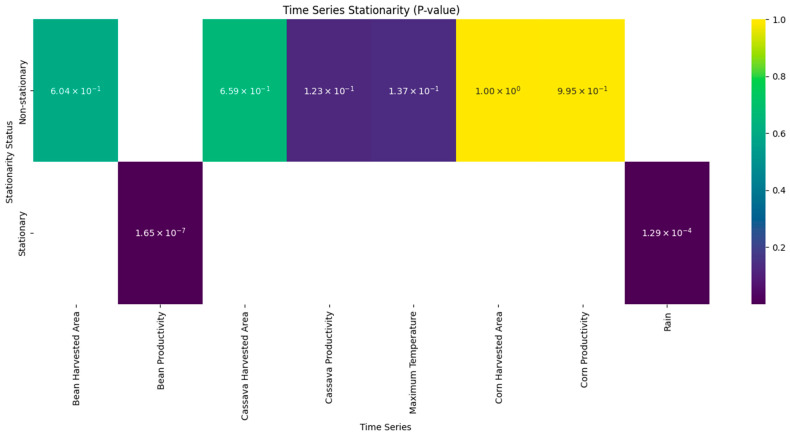
Results of the ADF stationarity test for each data series. Colors indicate *p*-value variation.

Figure 4 shows the results of the stationarity test for the series. The results show that only bean productivity (*p* ≈ 1.65 × 10^−7^) and annual precipitation (*p* ≈ 1.29 × 10^−4^) show statistically significant stationarity at the 5% level. This indicates that both variables fluctuate around a stable average, with no persistent growth or decline trend. In the case of bean productivity, this characteristic suggests that, although there are annual fluctuations due to factors such as pests, diseases, or management, the series maintains structural stability over the period.

Similarly, annual precipitation is stationary, corroborating the idea that, even with interannual variations linked to climatic phenomena such as El Niño or La Niña, the region’s rainfall regime does not show a significant long-term trend. However, the other variables analyzed—area harvested for beans (*p* ≈ 0.604), area harvested for cassava (*p* ≈ 0.659), cassava productivity (*p* ≈ 0.123), maximum temperature (*p* ≈ 0.137), corn harvested area (*p* ≈ 1.00), and corn productivity (*p* ≈ 0.995)—do not reject the null hypothesis of the presence of a unit root and are therefore non-stationary.

**Figure 5:**
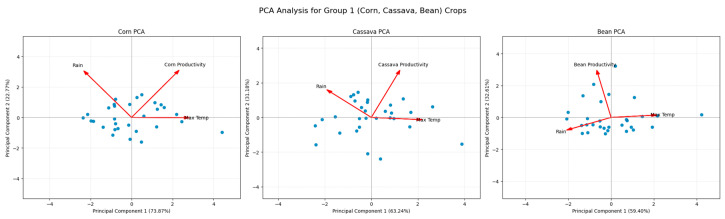
Principal component analysis for annual crops (corn, cassava, and beans).

Figure 5 shows the PCA for bean, corn, and cassava crops. In the case of corn, corn productivity is strongly associated with the first principal component (PC1), which explains 73.87% of the variance. The orientation of the vector indicates that both productivity and maximum temperature contribute significantly to this axis, while precipitation is positioned in the opposite direction. This pattern suggests that warmer years tend to be related to higher corn productivity, possibly due to crop management or physiological tolerance, although the negative influence of rainfall points to potential limitations due to excess water. For cassava, the first principal component (63.24% of variance) shows a direct association between cassava productivity and rainfall, with an orientation opposite to that of maximum temperature. This reinforces the critical role of water availability in cassava performance, a crop traditionally adapted to tropical conditions but still sensitive to water stress. High temperature appears as a potentially limiting factor, suggesting that warming scenarios may compromise productivity gains, especially if accompanied by irregular rainfall.

For beans, the first two components explain 59.40% and 32.61% of the variance, respectively, totaling more than 90%. The analysis indicates a strong positive association between bean productivity and rainfall, while maximum temperature contributes in the opposite direction. This pattern is consistent with agronomic knowledge that beans are highly sensitive to both water deficiency and high temperatures, which negatively affect flowering and grain filling. The PCA confirms the predominant role of precipitation in explaining the variability of bean productivity.

**Figure 6:**
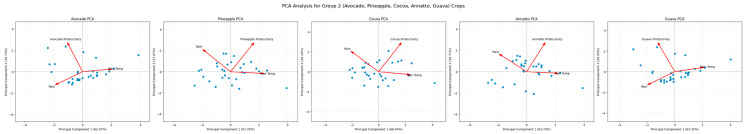
Principal component analysis for annual crops (pineapple, avocado, cocoa, annatto, guava).

Figure 6 shows the PCA for avocado, pineapple, cocoa, annatto, and guava crops. In the case of avocado, the first principal component (62.07%) is marked by a strong association between productivity and maximum temperature, while precipitation appears to be oriented in the opposite direction. This result suggests that avocado productivity is favored by higher temperatures but may be limited by excessive precipitation.

For pineapple, PCA shows that productivity correlates simultaneously with rainfall and temperature, explained mainly by the first component (67.76%). This pattern reinforces the crop’s sensitivity to both water availability and temperature, indicating that extreme weather conditions in either direction (droughts or high temperatures) can negatively affect production.

In the case of cocoa, a similar association is observed, with productivity positively correlated with both rainfall and maximum temperature, explained largely by the first component (68.04%). This result is consistent with the physiological profile of the crop, which is highly dependent on precipitation but also responds positively to adequate temperatures within tolerable limits.

The analysis of annatto indicates that its productivity is aligned with rainfall, while maximum temperature acts in the opposite direction. The first component (63.79%) highlights this dichotomy, suggesting that the crop particularly benefits from adequate rainfall regimes but suffers negative impacts from high temperatures. Regarding guava, PCA shows a strong association between productivity and maximum temperature, similar to the pattern observed for avocado, with the first component (63.35%) reflecting the dominance of this climatic variable. Precipitation appears to have a secondary and contrary influence, indicating that excessive rainfall can impair productive development.

The results show that cocoa cultivation had the largest expansion in harvested area among all those analyzed, which can be explained by the significant increase in the value of this commodity over the last year, as shown in Table 1.

## 4. Discussion

The results obtained show that climate variability, especially the progressive increase in maximum temperature between 1994 and 2023, exerts different pressures on perennial and annual crops. Although annual precipitation does not show a clear trend of change over the period, the rise in temperature, which exceeds 31.5 °C in recent years of the series, is a critical climatic factor, in line with global warming projections [2]. The maximum rainfall in the series (2350 mm) occurred in 1999, classified as a strong La Niña year [46], as well as 2011 (2334 mm), the second wettest year in the series, which is also characterized as a strong La Niña year by the same reference. The driest year in the series is 2023 (1738 mm), known to be the hottest and driest year in history due to the action of a super El Niño [47].

The driest years are 2015 (1947 mm) and 1998 (2018 mm), also widely recognized in the literature as years of intense action by an El Niño phenomenon that drastically reduced rainfall in the north of the country [48]. Avocado production in the northern region has declined sharply since 1999 (maximum harvested area 1293 ha in 1998 to 78 ha in 2023), in contrast with the global trend of expansion [49]. This is consistent with projections by Grüter et al. [50], which indicate that climate suitability for avocado will decrease in Brazil due to rising temperatures. Beyond climate stress, economic drivers also explain this reduction, as farmers shift land use to more profitable crops such as cocoa and corn.

In addition, for economic reasons, it is possible that rural producers in the northern region are allocating plantation areas to other, more economically attractive monocultures, which would explain this decrease in production and planted area over time. Cocoa showed steady expansion in planted and harvested areas, largely sustained by technological improvements and irrigation. Although some studies suggest high sensitivity to water deficits [51], our results show resilience in production despite rainfall variability. Similar patterns were reported by Igawa et al. [52], who warn that long-term climate change could still reduce cocoa suitability in the Amazon by 2050. Other studies have also shown that there is greater stability in cocoa cultivation and better environmental outcomes with the adoption of Agroforestry Systems (AFS) [53], which makes it attractive for producers to invest in this monoculture, since cocoa agroforestry systems in the Amazon demonstrate sustainability and productivity.

There has been a noticeable downward trend in the area planted for specific monocultures of less economic attractiveness in the north of the country in recent years [54,55], which is linked mainly to the increase in areas for planting other commodities, such as corn and soybeans. The years with a tendency for increased rainfall also favor increased productivity in the bean crop, as evidenced in the years 1999, 2003, 2006, 2017, and 2021, which, in a scenario of reduced rainfall due to climate change, should cause a reduction in the productivity of this crop.

In contrast, corn production expanded almost fivefold after 2015, with little correlation to climate, reflecting technological intensification and global demand. Pineapple and guava displayed relative resilience, with production driven more by agronomic practices than by rainfall variability [55,56]. This suggests that technological inputs may buffer climate impacts for some fruit crops, but only when market incentives are present. There are studies indicating that climate change may be beneficial for certain specific crops, such as corn, which could see increased production in states like Amazonas, Roraima, and Amapá [57], which supports our findings.

In the Amazon, even when it comes to pineapple production via family farming, there is a technological insertion in the area aimed at the market [55], which may explain the production’s high resilience to variations in the local climate. Although it has shown little sensitivity to climate change, the impact of climate change on guava production cannot be dismissed, as an increase in air temperature could have long-term implications, such as changes in the phenological cycle or greater susceptibility to heat stress [56].

In the case of cassava, although production fell drastically (from more than 8 ts/year to 5 t/year) between 2014 and 2023, the tendency for rainfall to increase between 2019 and 2021 coincides with a slight tendency for production to increase, showing greater vulnerability to environmental changes. Numerical experiments have simulated cassava production in different regions of the world, considering future climate scenarios with changes in temperature, precipitation, and CO_2_ concentration [58,59,60,61].

In addition to climate issues, the food security of traditional communities, indigenous peoples, quilombolas, and riverine communities may also be affected by the shift from the production of everyday food products, such as cassava, to monocultures that have been exploited to supply the foreign market through exports. Cassava, which was previously the main agricultural product in the state of Pará, has been replaced by açaí [62], which has seen a dramatic increase in export volumes, leading to an “açaíization of the landscape” in the Amazonian floodplains [63]. This may also be happening with cocoa in other regions of the Amazon, taking into account the results of this article, thus causing a “cocoaization of the landscape”, as a result of several factors, such as population pressure, the search for natural resources, the rise of the capitalist model, technological development, land values and the expansion of the agricultural frontier [64].

This crop has expanded its cultivated area significantly, indicating incentive policies and economic appreciation [65]. Pineapple and annatto crops, on the other hand, have relatively stable productivity, but with a slight decline in recent years. This variability may be related not only to climate but also to agricultural management practices, use of technologies, and incidence of pests and diseases [66].

Using a statistical agrometeorological model and 20 general circulation climate models (GCMs) of the CMIP3 generation, Lobell et al. (2008) [59] investigated the production of crops important for food security in 2030. For Brazil, the simulation indicated a 5% drop in cassava productivity due to rising temperatures and reduced rainfall, an effect that is corroborated by the data shown here. The corn production in the northern region of Brazil is influenced by a combination of climate variability and socio-economic factors. While extreme weather events, such as droughts associated with El Niño, directly impact productivity, issues such as poor infrastructure and regional inequality exacerbate the challenges faced by producers.

Integrated approaches that consider both environmental and socio-economic aspects are essential to promote sustainable and resilient agriculture in the region. Areas such as the south of the state of Pará, in particular, have experienced an expansion of the agricultural frontier, known as the “arc of deforestation”. This expansion has been driven by the search for new areas for cultivation, resulting in an increase in the area planted and harvested for corn, as can be seen in the graph from 2016. The expansion of Brazilian corn production in recent years has been sustained mainly by international demand for the cereal, which has absorbed the domestic surplus.

Looking at the last nineteen crop years, between the 2000/2001 and 2018/2019 harvests, while domestic consumption increased by 86%, national corn production increased by 137% [63]. The growing international demand for corn has positively influenced prices in the Brazilian market, encouraging producers to expand their cultivation areas. Between the 2000/2001 and 2018/2019 harvests, national corn production increased by 137%, while domestic consumption grew by 86%, highlighting the role of exports in this growth [67,68].

Concerning perennial crops, greater interannual variability in productivity was observed, notably in avocado and guava, while pineapple remained relatively stable, and cocoa showed fluctuations offset by the expansion of the harvested area. This instability suggests a strong influence of environmental and phytosanitary factors, since these species remain in the field for long periods and accumulate the effects of thermal and water stress over the years. Furthermore, the vulnerability of these crops may be associated with low incorporation of technological innovations, dependence on traditional practices, and greater exposure to pests and diseases favored by warming [69].

The production of annatto is a practice characteristic of traditional family farms or settlements, and has contributed to keeping people in the rural areas, demonstrating the social importance of cultivation [70]. The peak of Urucum production was in 2018, and it has been declining ever since, until the minimum seen in 2022. It is well known that the crop is greatly influenced by the climate [5], developing well at an air temperature of 22 and 27 °C [71]. Studies show that from 2081 onwards, the situation of annatto production in Brazil will become critical, with more than 90% of the producing areas presenting unsuitable conditions due to excess heat. Around 80.08% of the national territory has been classified as unsuitable for production, 79.95% of which is due to excess heat, which represents an increase of 71.71% compared to the current situation [72].

Beans and cassava are highly sensitive to rainfall anomalies, as observed during El Niño years (1998, 2005, 2015, 2023). These findings align with Liu et al. [58], who projected productivity losses under warmer and drier scenarios. Urucum production also shows a marked dependence on rainfall and is projected to become unsuitable in most Brazilian regions after 2080 [71]. This reinforces the vulnerability of staple crops crucial for regional food security.

The fact that the area planted with Urucum has fallen by around ¼ since 2002 is noteworthy, highlighting the loss of area for planting commodities, such as cocoa [73]. The data in Table 1 shows the probable reason for this, given the high value of cocoa on the market today compared to other crops, which can lead to areas being set aside for cocoa plantations to the detriment of the production of essential foods or spices that are widely used by the population, as was observed with beans, cassava, and annatto.

Comparing the two groups, it is noted that perennial crops are more susceptible to long-term climate fluctuations [74], presenting high interannual variability in productivity, while annual crops are able to respond more quickly through technological innovations, even though they have limitations in the face of continuous warming. Thus, while perennial-based systems face increasing risks of instability, annual systems demonstrate greater, but not unlimited, resilience.

Statistical analyses indicate that only a few climatic and production variables show long-term stability, while most are subject to continuous structural changes. This reinforces the hypothesis that agriculture, especially in the tropical context, is strongly conditioned by external and dynamic factors, requiring integrated analyses that combine advanced statistics, climate science, and agricultural economics. This result reveals that these series are subject to persistent trends, possibly associated with structural factors such as agricultural policies, technological changes, economic pressures, and, above all, climate change. The non-stationarity of maximum temperature is particularly relevant, as it suggests a gradual increase over recent decades, consistent with the effects of global warming reported in several climate studies [75].

Similarly, the areas harvested for cassava and corn reflect land use dynamics influenced by economic and social decisions that transcend natural variability [76]. Cassava and corn productivity, though not stationary, may indicate technological advances in certain periods, followed by stagnation or decline, so that the long-term pattern does not stabilize.

Corn cultivation, which has such a low return (Table 1) compared to beans, for example, has experienced a dramatic increase in harvested area, probably due to its various uses, ranging from the manufacture of feed for cattle, pigs, and poultry to the production of biofuels such as ethanol [77], in addition to other domestic uses. Associated with this, government incentives and advances in management technologies, such as genetic improvement and the use of irrigation, contribute to the increase in harvested area year after year in the Amazon, following the trend in the rest of Brazil [77].

Other studies [76] also demonstrate how the growth in commodity production, including corn, has been the result of both an increase in area (agricultural frontier) and technological changes, the displacement of pastures for cultivation, etc. The results show that economic/institutional decisions contribute to the expansion or contraction of cultivated areas.

Overall, the PCA results for annual crops reveal that while corn is more climate-resilient and positively associated with temperature, cassava and beans are more dependent on water availability and vulnerable to warming. This implies the importance of differentiated management strategies for each crop in the face of climate change, such as the use of adapted varieties, water conservation practices, and adjustments to the agricultural calendar.

The PCA results for the perennial crop group highlight a dual pattern: while some crops, such as avocado and guava, show greater affinity for high temperatures, others, such as annatto and pineapple, demonstrate a more direct dependence on precipitation. Cocoa, in turn, shows a balanced response to both variables. These findings reinforce the need for differentiated and regional management strategies, especially in a scenario of climate change, where increased rainfall variability and global warming may impact each crop analyzed differently.

The economic interest in increasing the production of certain commodities causes, among other problems, a severe change in the Amazonian landscape, with floristic impoverishment [78], a large reduction in the diversity of food sources for local populations, and financial dependence on the exploitation of a single or few natural resources which, if affected by disease, pests, or extreme weather conditions, cause losses that can be irreversible for those who depend solely on them for their subsistence, requiring public policies that by disease, pests, or extreme weather, can cause irreversible damage to those who depend solely on them for their livelihood [79]. This requires public policies that seek to support producers in becoming resilient to environmental changes and increase the variety of their income sources, thereby strengthening the local bioeconomy [80].

## 5. Conclusions

The results showed that climate variability influences annual and perennial crops differently. While corn showed gains associated with temperature, crops such as beans and cassava showed greater dependence on precipitation, revealing vulnerability to periods of drought. In the perennial group, avocado and guava responded positively to high temperatures, while annatto and pineapple maintained a greater relationship with water availability.

Cocoa shows consistent growth in production, as does corn, and this growth is associated with technological advances and expansion of planted areas, although they are sensitive to extreme heat. Pineapple, however, shows a lower correlation with variations in rainfall, suggesting that agricultural management factors play a greater role. Guava shows a stability in production, even in climatically adverse years, indicating a greater influence of agricultural practices on productivity.

Nevertheless, the continued rise in temperatures could bring future challenges, such as greater susceptibility to heat stress. These results highlight the need for integrated strategies that consider climate and economic impacts for the sustainability of agriculture in Brazil’s northern region. Investments in agricultural technologies, resilient practices, and efficient management are essential to mitigate the effects of climate change and ensure the viability of these crops in the future.

Our findings demonstrate that staple crops such as cassava, beans, and annatto are highly vulnerable to drought and rising temperatures, while commodity crops such as cocoa and corn show resilience due to technological intensification and market incentives. These contrasting trajectories highlight a growing risk to regional food security in the Amazon. Policy implications include the urgent need to support smallholder farmers with access to irrigation, bio-inputs, and credit, while promoting diversified farming systems that combine food security with bioeconomy value chains. Incentives for agroforestry, tax relief for sustainable practices, and targeted programs for resilient crop varieties should be prioritized. This study has some limitations, including reliance on secondary datasets (IBGE and ERA5 reanalysis) and the absence of fine-scale socio-economic data. Future research should combine climate modeling with household-level surveys to capture both environmental and social dimensions of food security in the Amazon.

Considering this scenario, adaptation strategies are essential. For perennial crops, it is recommended to adopt agroforestry systems, use varieties that are more resistant to biotic and abiotic stresses, and implement integrated pest management. For annual crops, continued technological innovation must be accompanied by measures to mitigate the impacts of heat, such as efficient irrigation, crop rotation, and the development of varieties that are tolerant to drought and high temperatures.

These findings reinforce that agricultural adaptation must consider the specific characteristics of each crop, integrating practices such as the selection of tolerant varieties, efficient irrigation, and agroforestry management. Therefore, public policies aimed at climate resilience and investments in technological innovation are essential to sustain agricultural productivity in the face of global warming.

## Figures and Tables

**Table 1:** Prices of bioeconomy products analyzed in this article, in USD/t.

Product	Price in 2024 (USD/t)	Source
Cocoa	7390	[38]
Avocado	2640	[39]
Guava	2873	[40]
Beans	982	[41]
Pineapple	930	[42]
Cassava	1450	[43]
Annatto	385 (estimate)	[44]
Corn	202	[45]

## Data Availability

The original contributions presented in the study are included in the article; further inquiries can be directed to the corresponding author.
